# An Evaluation of Human Induced Pluripotent Stem Cells to Test for Cardiac Developmental Toxicity

**DOI:** 10.3390/ijms22158114

**Published:** 2021-07-29

**Authors:** Lauren Michelle Walker, Nicole R. L. Sparks, Veronica Puig-Sanvicens, Beatriz Rodrigues, Nicole I. zur Nieden

**Affiliations:** Stem Cell Center and Department of Molecular, Cell & Systems Biology, College of Natural and Agricultural Sciences, University of California Riverside, Riverside, CA 92521, USA; lwalk006@ucr.edu (L.M.W.); nicoles@ucr.edu (N.R.L.S.); puigsanv@oregonstate.edu (V.P.-S.); Beatriz.rodrigues@ucr.edu (B.R.)

**Keywords:** induced pluripotent stem cells, cardiomyocytes, embryotoxicity screen, tobacco smoke solution, embryonic stem cell test

## Abstract

To prevent congenital defects arising from maternal exposure, safety regulations require pre-market developmental toxicity screens for industrial chemicals and pharmaceuticals. Traditional embryotoxicity approaches depend heavily on the use of low-throughput animal models which may not adequately predict human risk. The validated embryonic stem cell test (EST) developed in murine embryonic stem cells addressed the former problem over 15 years ago. Here, we present a proof-of-concept study to address the latter challenge by updating all three endpoints of the classic mouse EST with endpoints derived from human induced pluripotent stem cells (hiPSCs) and human fibroblasts. Exposure of hiPSCs to selected test chemicals inhibited differentiation at lower concentrations than observed in the mouse EST. The hiPSC-EST also discerned adverse developmental outcomes driven by novel environmental toxicants. Evaluation of the early cardiac gene *TBX5* yielded similar toxicity patterns as the full-length hiPSC-EST. Together, these findings support the further development of hiPSCs and early molecular endpoints as a biologically relevant embryotoxicity screening approach for individual chemicals and mixtures.

## 1. Introduction

The mammalian developmental process is a sensitive and highly-regulated period of life. During this time, developing mammalian organisms are subject to numerous and complex processes that are critical for proper formation. As such, exposure to some chemicals, pharmaceuticals, or other agents during particular pregnancy windows could result in adverse developmental outcomes such as growth retardation, structural and/or functional abnormalities, and/or embryo lethality. Current regulations require commercially available industrial chemicals and pharmaceutical products to be evaluated for developmental repercussions [1,2]. These screening approaches evaluate adverse pregnancy outcomes in relation to maternal toxicity to determine the overall embryotoxic specificity of the agent, if any. While traditional in vivo screening approaches offer a wealth of information regarding the developmental toxicity of an agent, exclusively animal-based screens are time-consuming, expensive, and require a large number of animals to complete statistical evaluations. Perhaps most critically, exclusively animal-based evaluations carry the risk of false negatives due to species variations. The collective challenges of animal models have galvanized the growing momentum of focus on in vitro replacements for toxicity screens and studies.

Early iterations of in vitro developmental toxicity assays employed a variety of cell and tissue cultures, including primary embryonic cell cultures and whole mammalian embryos to determine general embryotoxicity and specific malformations, respectively [3]. The establishment of mouse blastocyst-derived pluripotent embryonic stem cells (ESCs) [4], however, revolutionized in vitro toxicity screen approaches. Given their unspecialized nature, pluripotent ESCs can recapitulate the developmental process in vitro through directed differentiation into particular cell types. Spielmann and colleagues capitalized on this capacity of mouse ESCs (mESCs) in their development of the mouse embryonic stem cell test (mEST). The mEST is an in vitro battery of assays designed to evaluate embryotoxicity without routine animal sacrifice [5]. In the mEST protocol, mESCs are differentiated into contracting cardiac muscle cells while simultaneously exposed to the agent under evaluation. Effects on ESC viability and differentiation capacity are also compared to 3T3 fibroblast cells to mimic maternal tissues and identify maternal toxicity that could secondarily influence embryotoxicity outcomes. Results from all three endpoints are ultimately entered into a biostatistical prediction model to determine and rank the embryotoxicity of the agent as non-embryotoxic, weakly embryotoxic, or strongly embryotoxic [6,7,8].

During its initial inception, accuracy of the mEST biostatistical model was verified using known positive (5-FU, *at*RA) and negative (PenG) control chemicals to confirm consistent classification of non-, weak, and strong embryotoxicants [7,8]. While the success of the mEST is best captured by its ability to reduce animal sacrifices in toxicity screens, it is still limited by the challenge of species–species variation in toxicity screens and mechanistic studies. Our group previously showed that non-human primate ESCs showed differential sensitivity to assorted classes of compounds compared to mouse ESCs [6]. Our results highlighted the analytical limitations introduced by species variation and highlight the need for robust methods that are as biologically relevant as possible to humans.

While their reported use in developmental toxicity assessments is currently limited, human pluripotent stem cell lines may be the ideal solution to species-based differential sensitivity by providing a relevant genetic and proteomic environment for toxicity assessments. For instance, a recent comparison in mouse versus human biochemical response to teratogenic thalidomide revealed that thalidomide targets SALL4 protein in human but not rodent cells [9]. Thus, employment of human cells in embryotoxic evaluations may prove more efficacious in determining embryotoxicity. Particularly promising candidates are human induced pluripotent stem cells (hiPSCs) as such lines can be induced to differentiate like ESCs, without the potential ethical or legislative challenges presented by routine use of human ESC lines. hiPSCs are already routinely used as in vitro models of human cardiotoxicity and disease [10,11], though reported use of hiPSCs in in vitro embryotoxicity studies is limited.

In this study, we seek to assess hiPSC utility in the EST protocol using the original evaluation scheme on the above mentioned positive and negative controls of the original cardiac mEST. Moreover, we test the efficacy of our hiPSC-EST protocol in a proof-of-concept embryotoxicity screen using tobacco product extracts.

## 2. Results

### 2.1. hiPSCs Efficiently Differentiate into Cardiomyocytes

**Figure 1:**
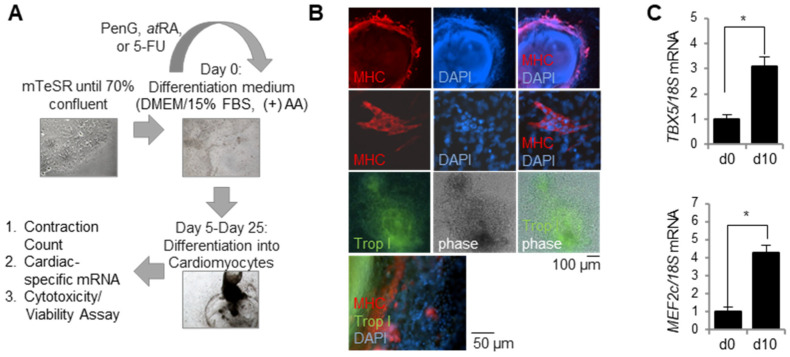
hiPSCs consistently and robustly differentiated into cardiomyocytes. Differentiated cardiomyocytes were assessed for cardiomyocyte-specific markers and gene expression. (**A**) Schematic protocol for cardiomyocyte differentiation and embryotoxicity screen. (**B**) Immunocytochemistry stains of differentiated cultures confirmed cardiomyocyte identity via myosin heavy chain (MHC) and Troponin I (Trop I). (**C**) Differentiated cardiomyocytes expressed cardiac-specific genes *TBX5* and *MEF2c* as measured by qPCR, *n* = 3 biological replicates ± SD, * *p* < 0.05, Student’s *t*-test. hiPSC, human induced pluripotent stem cell; MHC, myosin heavy chain; Trop I, Troponin I.

Prior to testing the efficacy of the hiPSC-based EST, RIV9 hiPSC cultures were evaluated for their cardiac differentiation potential and efficiency following a 25 day differentiation protocol (Figure 1A). Successful differentiation into cardiomyocytes was visually confirmed with the generation of active contractile clusters on days 20 and 25 of differentiation. The cardiac identity of these clusters was confirmed molecularly via mature cardiomyocyte markers. Immunocytochemistry stains of differentiated cell cultures on day 25 revealed positive staining for myosin heavy chain (MHC) and Troponin I (Trop I) in contractile clusters (Figure 1B). Moreover, RT-PCR analysis of cardiac-specific transcription factors at differentiation day 10 showed a significant upregulation of *TBX5* and *MEF2c* in hiPSC cultures induced to a cardiac cell fate (Figure 1C). Together, these results indicated that RIV9 hiPSCs successfully and efficiently differentiate into cardiomyocytes under the appropriate culture conditions.

### 2.2. The hiPSC-Based EST Determines Accurate Assessment of Embryotoxic Compounds

To verify the utility of hiPSCs in the validated EST protocol, cardiogenic differentiation was induced in hiPSC cultures with concurrent treatment of one of the following compounds used in the original EST protocol evaluations: PenG (non-toxic), 5-FU (cytotoxic) and *at*RA (embryotoxic). Over the course of the differentiation, hiPSC-derived cardiomyocytes were then subjected to visual quantitation of active contractile cells and clusters on days 15, 20, and 25. At the conclusion of the differentiation, cultures were also evaluated for changes in cell viability.

**Figure 2:**
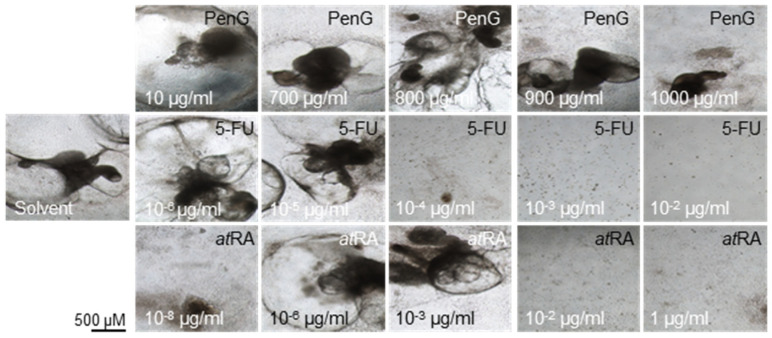
Presence of contractile cardiomyocyte clusters declines with treatment with embryotoxicants 5-FU and *at*RA. hiPSCs were treated with different concentrations of 5-FU, *at*RA, or PenG and photographed. hiPSC, human induced pluripotent stem cell; 5-FU, 5-fluorouracil; *at*RA, all-trans retinoic acid; PenG, penicillin G.

**Figure 3:**
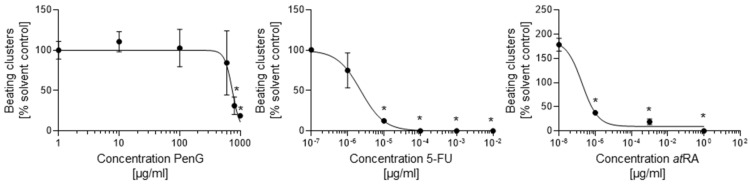
Treatment with embryotoxicants 5-FU and *at*RA impeded cardiomyocyte differentiation. hiPSCs were treated with different concentrations of 5-FU, *at*RA, or PenG and evaluated for differentiation inhibition by visually scoring the number of actively contracting cardiomyocyte clusters. Each data point represents the mean of three independent experiments ± SD. *: *p* < 0.05 = the lowest concentration significantly below the untreated control group as determined by One-Way ANOVA. hiPSC, human induced pluripotent stem cell; 5-FU, 5-fluorouracil; *at*RA, all-trans retinoic acid; PenG, penicillin G.

While no actively contracting cells or clusters were observed on day 15 of differentiation for any treatment group, differences in contraction incidence were readily observed between days 20 and 25 of differentiation for 5-FU- and *at*RA-treated cultures compared to the untreated control. 5-FU exposure negatively impacted the formation of contractile structures at concentrations above 1 × 10^−6^ μg/mL (Figure 2) by day 20. 5-FU exposure further returned a half-maximal inhibitory dose (ID_50_) for differentiation of 2.29 × 10^−6^ μg/mL on day 25 (Figure 3) (Table 1).

*at*RA-treated cells failed to form contractile clusters at concentrations above 1 × 10^−3^ μg/mL (Figure 2) and exhibited significantly reduced contractile activity in doses above 1 × 10^−8^ μg/mL (Figure 3). The resulting ID_50_ value for *at*RA on day 25 was 9.33 × 10^−7^ μg/mL. In contrast, treatment with PenG did not inhibit the formation of contractile structures (Figure 2) and did not negatively impact contractile activity at most of the tested concentrations (Figure 3). Dips in contractile incidence were only observed at doses beyond 700 μg/mL. An ID_50_ was determined at 739.9 μg/mL.

**Figure 4:**
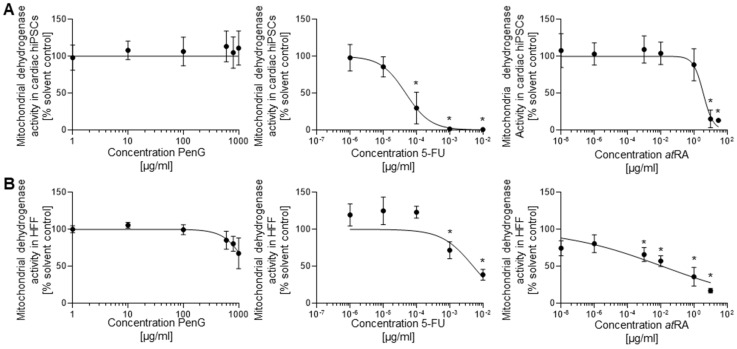
5-FU and *at*RA treatment reduced hiPSC-cardiomyocyte and hFF viability in a dose-dependent manner as assessed via MTT assay. (**A**) hiPSC viability screens for 5-FU, *at*RA, and PenG, *n* = 3 biological replicates ± SD. *: *p* < 0.05 = the lowest concentration significantly below the untreated hiPSC control group as determined by One-Way ANOVA. (**B**) hFF viability screens for 5-FU, *at*RA, and PenG, *n* = 3 biological replicates ± SD. *: *p* < 0.05 = the lowest concentration significantly below the untreated hFF control group as determined by One-Way ANOVA. hiPSC, human induced pluripotent stem cell; MTT, mitochondrial dehydrogenase activity assay; 5-FU, 5-fluorouracil; *at*RA, all-trans retinoic acid; PenG, penicillin G; hFF, human foreskin fibroblast.

However, cell viability assessments did not find any reduced cell survival in PenG-treated cells (Figure 4A) in the tested concentration range, suggesting the half-maximal inhibitory concentration for cell viability (IC_50_) value for PenG was above 1000 μg/mL.

In contrast, 5-FU treatment produced significant reductions in cell viability at concentrations above 1 × 10^−6^ μg/mL (Figure 4A) and returned an IC_50_ of 4.726 × 10^−5^ µg/mL. Because 5-FU-driven reductions in cell viability were observed around the same doses at which contractile activity was inhibited, these results together suggest that the inhibitory impact of 5-FU may primarily be driven by cytotoxic activity. *at*RA-treated cultures only displayed reductions in cell viability at the highest tested concentrations of 1 and 100 µg/mL (Figure 4A). Unlike 5-FU, *at*RA-driven inhibition of differentiation occurred at much lower concentrations than where cytotoxicity was observed. This outcome indicates that *at*RA may operate predominantly through an embryotoxic mechanism that inhibits differentiation without conveying outright cytotoxicity. The IC_50_ value for *at*RA was 3.642 µg/mL (Table 1).

The impact of the tested compounds in differentiating hiPSCs was also compared against treatment of differentiated hFF cells to determine embryotoxic specificity (Figure 4B). Both 5-FU- and *at*RA-treated hFF cells demonstrated a dose-dependent reduction in cell viability. Significant reductions were observed for 5-FU and *at*RA above concentrations of 1 × 10^−4^ μg/mL and 0.01 μg/mL, respectively. Dosing hFF cultures with 5-FU returned an IC_50_ value of 8.079 × 10^−4^ μg/mL, which was larger than the IC_50_ value produced by hiPSC-cardiomyocyte evaluations. This result suggests the general cytotoxicity of 5-FU, while supporting that differentiating hiPSC-derived cardiomyocytes are notably more susceptible to 5-FU toxicity than differentiated hFF cells. hFF cells treated with *at*RA experienced significant cell death at the highest tested concentration of 1 μg/mL. *at*RA-dosed hFFs produced an IC_50_ value of 1.247 × 10^−2^ μg/mL, which was higher than the IC_50_ value found in the hiPSC-cardiomyocyte assessment. PenG-treated hFF cultures started to display a reduction in cell viability at 800 µg/mL with an estimated ID_50_ of 1433 µg/mL.

To classify compounds under evaluation, the validated EST applies a biostatistically based prediction model to classify compounds as non-embryotoxic, weakly embryotoxic, or strongly embryotoxic based on differentiation and cell viability assay outcomes. The prediction model uses a series of equations to perform a linear discriminant analysis using determined IC_50_ and ID_50_ values determined from dose–response curves [15]. Using this model with the results of the hiPSC-EST resulted in the accurate classification of PenG as non-embryotoxic and 5-FU and *at*RA as strongly embryotoxic (Table 1) as also initially noted in the validated mouse EST [14,16]. Importantly, half-maximal concentrations found for 5-FU and *at*RA were decades lower than the maximum plasma concentration (Cmax) achieved during human exposure underscoring the strong potential of these two compounds to elicit embryotoxic outcomes in vivo. Higher ID_50_ values observed in the mESC-EST suggest some resistance to the toxic effects of both chemicals in mESC cultures compared to hiPSCs. hFF response to the tested chemicals also appeared comparatively more sensitive than 3T3 cell viability outcomes, with lower IC_50_ values following 5-FU and *at*RA treatment. Thus, compared to the mESC-based EST, the hiPSC-based EST suggested a potential higher sensitivity compared the mouse cell-based response at least for the three chemicals tested. 

### 2.3. hiPSC-EST Yields Accurate Early Toxicity Classifications Using Molecular TBX5 mRNA Expression

One of the main critiques of traditional developmental toxicity evaluations is the length of time and technical training required to successfully complete assays. As such, a shorter, quantifiable, and correct in vitro embryotoxicity assessment could improve throughput without sacrificing accuracy. Given the correct predictions achieved with contraction counts described above, we next investigated if our approach could be modified with a shorter, qPCR-based endpoint to determine differentiation inhibition. Here, we selected two cardiogenesis-specific transcription factors, *TBX5* and *MEF2c* [17,18,19], to determine if the adverse differentiation outcomes would already be detectable as changes in *TBX5* and *MEF2c* expression on day 10 of differentiation.

**Figure 5:**
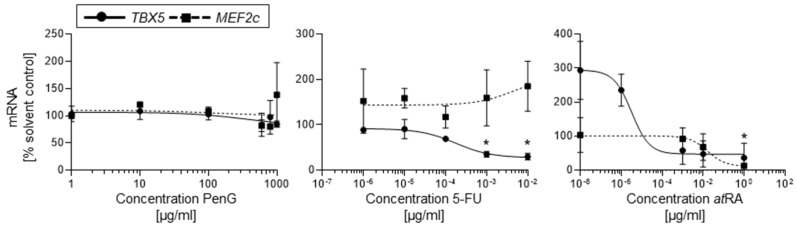
Treatment with embryotoxicants 5-FU and *at*RA impeded cardiomyocyte differentiation as measured by day 10 *TBX5* gene expression in hiPSCs. hiPSCs were treated with different concentrations of 5-FU, *at*RA, or PenG and evaluated for *TBX5* or *MEF2c* expression via qPCR. Data points represent means of three independent experiments ± SD. Inhibition of differentiation (ID_50_) was determined from dose–response curves as a 50% reduction in gene expression in the control. hiPSC, human induced pluripotent stem cell; 5-FU, 5-fluorouracil; *at*RA, all-trans retinoic acid; PenG, penicillin G. *: *p* < 0.05 = the lowest concentration significantly below the untreated hiPSC control group as determined by One-Way ANOVA.

Similar to the pattern observed in the contractile cluster assay, 5-FU-treated cultures demonstrated a dose-dependent reduction in *TBX5* mRNA expression with an ID_50_ value of 1.79 × 10^−4^ μg/mL (Figure 5). However, d10 *TBX5* mRNA expression was more dramatically reduced at concentrations above 1 × 10^−4^ μg/mL, compared to the lower reduction threshold of 1 × 10^−6^ μg/mL observed in the contractile assay. It is possible, however, that our day 10 analysis timepoint captured the beginnings of the full cytotoxic response that was able to fully manifest and be observed in cultures on d25 of differentiation. It follows, then, that embryotoxicants would be the most detectable at day 10 of differentiation in stronger concentrations with middle range doses producing a more moderate toxicity response.

Cells exposed to *at*RA also showed a dose-dependent downregulation of *TBX5* mRNA expression. In these cultures, exposure featured significantly downregulated *TBX5* beyond the determined ID_50_ value of 3.28 × 10^−6^ μg/mL (Figure 5). However, *at*RA treatment at tested concentrations lower than 1 × 10^−6^ μg/mL yielded a significant upregulation in *TBX5* mRNA expression. This upregulation correlated with the almost 2-fold increase in contractile cluster incidence seen in cultures treated with 1 × 10^−8^ μg/mL *at*RA (Figure 3). As retinoic acid is a well-reported regulator of *TBX5* expression in developing tissues, it is possible that exposure to low levels of *at*RA exposure may have encouraged *TBX5* mRNA expression [20] in differentiating hiPSCs, while *at*RA exposure above a particular threshold elicited an embryotoxic suppression of *TBX5*. Thus, the molecular response pattern in *TBX5* mRNA expression mirrored that observed in the contractile assay. Similar to 5-FU-treated cultures, it is possible that the early inhibitory impact of *at*RA at d10 of differentiation is readily observed via qPCR in middle and high concentration ranges. Treatment with the negative control PenG did not significantly impact *TBX5* mRNA expression (Figure 5). 

In contrast to expression patterns observed with *TBX5* mRNA, *MEF2c* mRNA expression yielded inconsistent responses to compound treatment. No reductions in *MEF2c* mRNA expression were observed for any 5-FU-treated group compared to the untreated control (Figure 5) and thus no ID_50_ value could be determined. In cells dosed with *at*RA, however, *MEF2c* mRNA transcripts were dose-dependently downregulated in differentiating cardiomyocytes (Figure 5). Here, a steady decline in *MEF2c* mRNA expression was observed at doses above 1 × 10^−3^ μg/mL with a final ID_50_ of 4 × 10^−2^ μg/mL. *MEF2c* mRNA expression in cells dosed with PenG remained largely unchanged from that of the untreated solvent control, though slight upregulation was observed at 800 and 900 μg/mL. Given the lack of altered *MEF2c* mRNA expression in cultures exposed to cytotoxic 5-FU compared to *at*RA, it is likely that *MEF2c* is an inconsistent indicator of early differentiation inhibition in cardiomyocytes. In contrast, *TBX5* mRNA expression patterns suggest that *TBX5* may be a better candidate for early differentiation inhibition assessment.

To compare the efficacy of qPCR-based embryotoxicity classifications versus contractile assay-based classifications, the ID_50_ values generated from 5-FU and *at*RA *TBX5* mRNA dose–response curves were compared against the ID_50_ values from the contractile assay curves in the EST biostatistical model. Both methods were found to produce the same embryotoxicity classifications for 5-FU, *at*RA, and PenG of strongly embryotoxic and non-embryotoxic, respectively (Table 2).

### 2.4. hiPSC-EST Suggests Embryotoxicity of Tobacco Solutions

5-FU and *at*RA were selected for verification of our hiPSC-based EST protocol, given their initial use in validation of the mESC-based EST and well-reported embryotoxicity. The observed correct classification builds a foundational rationale for testing the predictivity and sensitivity of this assay with additional chemicals. The true test of utility of the hiPSC-based EST, however, will be its ability to successfully evaluate novel embryotoxicants. To test how well the hiPSC-based EST evaluates novel embryotoxicants, the protocol was used to classify two different types of tobacco products: conventional cigarette smoke (Marlboro Red 100) and Snus smokeless tobacco (Camel Snus). Tobacco was selected as a test embryotoxicant as maternal smoking has previously been linked to a suite of negative effects on fetal development including low birth weight, congenital heart defects, and negative impact on bone growth and bone mass [21,22,23,24].

**Figure 6:**
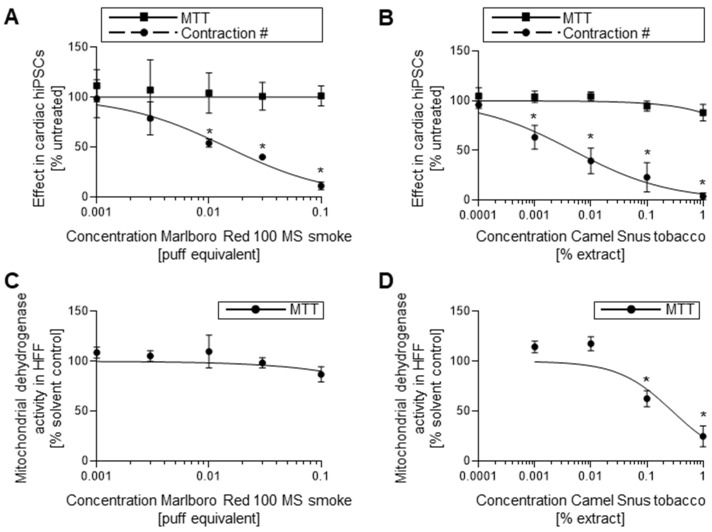
Effects of cigarette smoke and Snus smokeless tobacco on developing cardiomyocytes. (**A**) Contractile and viability screens for hiPSCs exposed to MS cigarette smoke using contractile and MTT assay, *n* = 3 ± SD. (**B**) Contractile and viability screens for hiPSCs exposed to Snus smokeless tobacco using contractile and MTT assay, *n* = 3 ± SD. (**C**) Viability screen for hFFs exposed to MS cigarette smoke using MTT assay, *n* = 3 ± SD. (**D**) Viability screen for hFFs exposed to Snus smokeless tobacco using MTT assay, *n* = 3 ± SD. hiPSC, human induced pluripotent stem cell; MTT, mitochondrial dehydrogenase activity assay; MS; mainstream; hFF, human foreskin fibroblast. *: *p* < 0.05 = the lowest concentration significantly below the untreated hiPSC control group as determined by One-Way ANOVA.

Contractile assays found dose-dependent reductions in the formation of active contractile clusters and structures for both Marlboro Red 100 mainstream (MS) smoke (Figure 6A) and Camel Snus (Figure 6B) extract. Contractile assay dose–response curves returned ID_50_ values for 0.014 puff equivalent (PE) and 0.0048% *w*/*v* Camel Snus extract. The viability of differentiating hiPSCs during concurrent tobacco exposure was not negatively impacted by either product in the tested concentration ranges. Viability of hFF cultures exposed to Marlboro Red 100 MS smoke and Camel Snus extract was reduced in a dose-dependent manner. Camel Snus hFF exposure returned an IC_50_ value of 0.2844% extract. An IC_50_ value for Marlboro Red exposed hFF cultures was not determined within the dose range under evaluation but was expected to occur at a dose above 0.1 PE.

hiPSCs failed to develop cardiomyocyte structures at sub-cytotoxic concentrations (Table 3) with regard to both hiPSC and hFF MTT outcomes, a result that collectively suggests that both products have embryotoxic characteristics. In fetal serum, nicotine concentrations can be as high 15.4 μM [25], placing the ID_50_ values found for these two products well within the range of the applicable dose. Indeed, the biostatistical evaluation determined both tobacco products to be strongly embryotoxic, in line with the known outcome of in utero exposure to tobacco smoke.

## 3. Discussion

Here, we have shown that hiPSCs can be induced to produce a robust and consistent cardiac differentiation model suitable for use in embryotoxicity screening assays. Using the biostatistical model, assay endpoints, and chemical agents used in the original EST protocol, this hiPSC-based EST model correctly classified the original EST test chemicals, 5-FU and *at*RA, as embryotoxic and test negative control, PenG, as non-embryotoxic. These results support that hiPSCs may be utilized in the EST protocol to incorporate translational relevance to embryotoxicity assessments; although additional analyses with multiple chemical classes are required to confirm improved assay sensitivity and predictivity in the hiPSC-based EST protocol over the mouse EST. Reductions observed in hiPSC-determined ID_50_ and IC_50_ values for three control chemicals already do suggest a potential for differences in assay sensitivity with hiPSCs compared to the mESC-EST. This could be driven by genetic or metabolic differences between the cell lines used in both studies. While studies comparing the metabolic efficiencies of mouse and human pluripotent stem cells have yet to be reported, differential cytotoxic sensitivity between human and mouse has been previously observed in neuroblastoma cell lines exposed to organophosphate insecticides [26].

The hiPSC-EST model presented here was also modified with the addition of molecular endpoints to shorten screening duration and reduce personnel strain. Two significant critiques of traditional developmental animal models are the required time to complete exposure/assessment routines as well as the need for specialized personnel training. While the in vitro hiPSC contractile and MTT assays require less hands-on time than a whole animal model, this approach is still challenged by assay duration and the requirement for specialized training to complete the contractile assay correctly. To address this limitation, we evaluated cardiac gene expression at day 10 of differentiation instead of quantifying contractile structures at day 25. A molecular endpoint was selected as qPCR supplies, equipment, and protocols are readily available in most laboratory environments conducting developmental and reproductive toxicity studies.

The differential response in expression between the two cardiac markers chosen for the qPCR protocol, *TBX5* and *MEF2c*, underscore the importance of carefully selecting tissue and timepoint specific markers for assessment in this screening model. Changes in *TBX5* expression patterns mirrored that of the contractile assay dose–response curve, conversely *MEF2c* expression patterns were not consistent between all of the tested chemicals. While *MEF2c* expression is specifically detectable in differentiating cardiomyocytes at day 10 of differentiation, additional studies by our group have found that *MEF2c* expression is highest on day 25 of differentiation [27]. Thus, it is possible that before day 25 of differentiation, *MEF2c* expression is not yet at a robust enough level of expression to generate a consistent dose–response curve in actively differentiating cells. It should be noted, however, that the qPCR-derived ID_50_ values still successfully yielded the same embryotoxicity classifications for 5-FU, *at*RA, and PenG as the contractile assay when calculated with the EST biostatistical model. Collectively, this suggests that the EST protocol may be successfully utilized with hiPSCs without having to sacrifice the speed of the original mEST protocol or take on specialized training to conduct the contractile assay. 

Given the correct classification of the test embryotoxicants by the hiPSC-EST, we also assessed the ability of our model to determine the embryotoxicity of life-style toxicants. Cigarette smoke and Snus tobacco extracts have been connected to adverse pregnancy outcomes following prenatal maternal exposure and are comprised of a mixture of potentially hazardous chemicals [28]. While Snus smokeless tobacco has also been linked to impaired embryonic development following use during pregnancy [22,23], it is also frequently advertised to women of reproductive age as a harm-reducing alternative to cigarettes. To date, the precise chemical or chemicals responsible for adverse pregnancy outcomes have yet to be completely identified thus rendering these extracts “unknown chemical mixtures” for our purposes. In the concentration ranges tested, the hiPSC-EST successfully identified ID_50_ values for both Marlboro Red and Camel Snus that were lower or near plasma nicotine concentrations measured in embryos of smoking mothers. In that our results harmonize with epidemiological studies that connect maternal tobacco use with embryonic congenital heart defects [29]. Furthermore, they also suggest that the main predictor of embryotoxicity risk may be the relationship between the measured concentration at which differentiation is inhibited to fifty percent and the actual observed exposure concentrations in humans. While this may potentially obviate the need to test all three EST parameters, the fact that the ID_50_ values were considerably lower than where cytotoxicity was observed ion both cell lines does suggest a molecular basis for the embryotoxicity of both tobacco products that operates through differentiation inhibition rather than cytotoxic effects on differentiating cardiomyocytes. This is important since embryotoxic outcomes may stem from adverse embryonic events causing aberrant development (i.e., malformations) or cytotoxicity (i.e., growth retardation), as well as maternal toxicity that yields embryo implantation issues, growth retardation or spontaneous abortion. Given this outcome, the hiPSC-EST model with its three parameters could plausibly be used for molecular follow up analysis to determine root causes of environmental toxicant-elicited embryotoxicity observed at the screening phase. Furthermore, considering that cigarette smoke is a mixture of over 5000 chemicals [30], our results suggest that the hiPSC-EST model is also suitable for assessing embryotoxicity of environmental toxicant mixtures in addition to screens of individual toxicants. This feature could prove useful in risk assessment applications where the developmental toxicity potential of commercial, industrial, and/or environmental chemical mixtures is sought.

## 4. Materials and Methods

### 4.1. Culture of Human Induced Pluripotent Stem Cells (hiPSCs)

The hiPSC cell line RIV9 was obtained from the Stem Cell Core at the University of California, Riverside. hiPSCs were seeded on Matrigel (Corning, Glendale, AZ, USA)-coated tissue culture plates and maintained in a pluripotent state in mTeSR^®^ medium (Stem Cell Technologies, Seattle, WA, USA). Cells were cultured under a humidified atmosphere of 5% CO_2_ at 37 °C and passaged for maintenance or to seed for experiments approximately every 5 days as previously described [31,32].

### 4.2. Cardiac Differentiation of hiPSCs

After cells reached 70% confluency (designated day 0), the medium was changed to control differentiation medium supplemented with 0.06 mg/mL ascorbic acid (Sigma-Aldrich, St. Louis, MO, USA) to induce cardiac differentiation [27]. Control differentiation medium was comprised of Dulbecco’s modified Eagle’s medium (DMEM with 4.5 g/L glucose, L-glutamine and sodium pyruvate; Corning) supplemented with 18% FBS (PAA), 0.8% penicillin/streptomycin (10,000 units/10,000 units, Gibco, ThermoFisher Scientific, Grand Island, NY, USA), 0.12% non-essential amino acids (NEAA; Gibco), and 0.1 mM β-mercaptoethanol (Gibco). Cultures were cultured in differentiation media for 25 days, starting from day 0.

### 4.3. Culture of Human Foreskin Fibroblasts

Human foreskin fibroblasts (hFF) were gifted from Dr. Derrick Rancourt (University of Calgary). hFFs were seeded onto 0.1% gelatin-coated tissue culture plates and maintained in Dulbecco’s modification of Eagle’s medium (DMEM with 4.5 g/L glucose, L-glutamine and sodium pyruvate; Corning cellgro) supplemented with 10% FBS (Atlanta Biologicals, Flower Branch, GA, USA) and 0.5% penicillin/streptomycin (10,000 units/10,000 units, Gibco).

### 4.4. Preparation of Toxicant Solutions

5-flurorouracil (5-FU), all-trans retinoic acid (*at*RA), and penicillin G (PenG) were selected from a subset of chemicals used in the original EST validation study [33,34]. 5-FU and *at*RA were selected as positive test compounds due to their established embryotoxic potential while PenG was used as a negative control. All chemicals were purchased from Sigma. Stock solutions were prepared in DMSO and filtered through a 0.2 micron Acrodisc^®^ PSF Syringe Filter (Pall Corporation, Port Washington, NY, USA), aliquoted into sterile microcentrifuge tubes and stored at −20 °C until use. Test chemicals were serially diluted to final concentrations in differentiation media. Cardiogenic cultures were treated with designated chemicals (or solvent only) through day 25 of differentiation and hFF cultures were treated with each compound for a 25 day duration period. Compounds were replenished with each medium change.

A University of Kentucky smoking machine was used to produce smoke extract solutions from commercially available conventional Marlboro Red 100 brand cigarettes as previously described [35,36]. The smoking machine took a 2.2 s puff of mainstream (MS) smoke every minute. Smoke solution concentrations were made in puff equivalents (PE), which are the number of cigarette puffs dissolved in 1 mL of medium. MS smoke solutions were produced by pulling 30 puffs of MS smoke through 10 mL of DMEM. Resulting 3 PE smoke extracts were filtered through a 0.2 micron Acrodisc^®^ PSF Syringe Filter (Pall Corporation, Port Washington, NY, USA), aliquoted into sterile microcentrifuge tubes, and stored at −80 °C until use. Serial dilutions were performed in differentiation medium to reach desired final exposure concentrations. Cardiogenic cultures were treated with smoke solutions through 25 days of differentiation. Smoke solutions were replenished with each medium change.

A 10% (*w*/*v*) Camel Snus extract was prepared as previously described [37]. Ten grams of Snus was added to 85 mL of DMEM and allowed to incubate at 37 °C for 2 h. This initial extract solution was centrifuged for 10 min at 4500× *g*. Supernatant from the first round of centrifugation was then centrifuged again at 13,000× *g* for 1 h. The resulting supernatant was collected and pH adjusted to 7.4. 15 mL of FBS was added to the pH-adjusted Snus extract to produce a 10% stock solution of Snus tobacco extract (STE). The stock solution was then sterile-filtered with a 0.22 μm vacuum filter system. STE was aliquoted into sterile microcentrifuge tubes and stored at −80 °C until use. Serial dilutions were performed in differentiation medium to reach desired final exposure concentrations. Cardiogenic cultures were treated with STE through 25 days of differentiation. STE was replenished with each medium change.

### 4.5. Immunocytochemistry

hiPSC-derived cardiomyocytes were washed with sterile commercially available 1× PBS (Gibco, ThermoFisher Scientific, Grand Island, NY, USA) and fixed with 4% paraformaldehyde (Sigma-Aldrich, St. Louis, MO, USA) 4 °C for 30 min. Fixed cultures were then washed three times with 1× PBS for 5 min. Cell membranes were permeabilized with 0.1% Triton X-100 in 1× PBS (ThermoFisher Scientific, Grand Island, NY, USA) for 15 min at room temperature before being washed three more times with 1× PBS for 5 min. Cultures were incubated in a blocking solution of 10% fetal bovine serum (PAA) and 0.5% bovine serum albumin (ThermoFisher Scientific, Grand Island, NY, USA) in 1× PBS for 1 h at room temperature. Primary antibody against mouse anti-Myosin Heavy Chain (MHC, ab15, 1:500, abcam, Burlingame, CA, USA) and/or rabbit anti-Troponin I (Trop I, sc-15368, 1:200, Santa Cruz Biotechnology, Dallas, TX, USA) was added directly to blocking buffer solution following the initial blocking period and allowed to incubate overnight at 4 °C. Cultures were washed with 1× PBS three times for 5 min prior to secondary antibody incubation. Cells were incubated with 20 μg/mL DAPI (4′-6-Diamidino-2-Phenylindole, Sigma, D9542), anti-mouse 546 conjugated florescent antibody (A10036, ThermoFisher Scientific, Grand Island, NY, USA) and/or anti-rabbit 488 conjugated fluorescent antibodies (A21206, ThermoFisher) for 2 h at room temperature. Cells were washed three times in 1× PBS to remove background from non-specific secondary antibody binding prior to imaging on a Nikon Eclipse Ti inverted fluorescence microscope (Nikon Inc., Melville, NY, USA).

### 4.6. Cardiac Viability Assay

Cardiomyocyte survival following concurrent exposure to each compound was evaluated by 3-[4,5-dimethylthiazol-2-yl]-2,5-diphenylterazolium bromide (MTT) assay from three independent differentiations each carried out in technical quintuplicate as previously described [38]. On day 25 of differentiation, cells were incubated with MTT (5 mg/mL) for 2 h at 37 °C. MTT supernatant was removed and replaced with a desorb solution of 0.7% SDS in 2-propanol. The absorbance of the solution was measured at 570 nm in an iMark™ microplate reader (Bio-Rad) with 655 nm as a reference wavelength. Here, mitochondrial dehydrogenase activity on the MTT in solution is directly proportional to a blue-purple product that is detected at 570 nm. Hence, a decrease in absorbance is interpreted as a direct measurement of any reduction in the number of viable cells [6,39,40].

### 4.7. Cardiac Contractile Assay

Contractile cardiac clusters and individual contractile, or “beating”, cells were counted and recorded on days 15, 20, and 25 of differentiation from 24 wells per biological replicate (set up from independent differentiations) as previously described [41]. Individual beating cells and beating cell clusters were cumulatively quantified between measurement time points. Untreated control cells served as a baseline for normal contractile incidence for each differentiation. Changes in beating incidence between treatment groups and days were reported as a percentage of beating incidence in solvent controls.

### 4.8. Real-Time Quantitative PCR (qPCR)

RNA was extracted from three wells per biological replicate using the protocol from NucleoSpin RNA II kit (Macherey-Nagel, Mountain View, CA, USA) and pooled. RNA from three independent differentiations was then quantified using a NanoDrop^®^ 1000 spectrophotometer (ThermoFisher Scientific, Grand Island, NY, USA) at 260 nm. 25 ng of total RNA was used as a template for cDNA synthesis with a mastermix including 5 µL 5× reaction buffer, 1.25 µL 10 mM dNTPs, 1.25 µL 400 U/µL RNase inhibitor, 0.1 µL 200 U/µL reverse transcriptase, 0.1 µL 3 µg/µL random primer, and 1.5 µL DEPC H2O for a total of 25 µL per reaction. 25 ng cDNA transcripts were used for quantitative polymerase chain reaction (qPCR) SYBR green technology on the MyiQ cycler (Bio-Rad). The reactions were setup for 10 min of denaturing at 94 °C (initial), followed by 40 cycles of denaturing at 94 °C, and annealing at 60 °C each 45 s. The n-fold expression in target samples was calculated with the ΔΔCT method by standardizing Ct values to GAPDH expression [42]. Primer sequences for human GAPDH were 5′-GAGTCAACGGATTTGGTCGT-3′ and 5′-TTGATTTTGGAGGGATCTCG-3′. Target genes were cardiogenic markers *TBX5* and *MEF2c*. Primer sequences for human *TBX5* were 5′-CTGGACACCCCTAAACTGGA-3′ and 5′-TCCCACAGAGCTGAACTCCT-3′ and primer sequences for human *MEF2c* were 5′-CCATTGGACTCACCAGACCT-3′ and 5′-AGCACACACACACACTGCAA-3′ [27].

### 4.9. Nicotine Measurements

Marlboro Red 100 MS and Camel Snus stock solutions were sent to Enthalpy Analytical (Irvine, CA, USA) for measurement of nicotine concentrations. 

### 4.10. Statistical Analysis

Half-maximal inhibitory compound doses of differentiation (ID_50_) and cytotoxicity (IC_50_) were determined from concentration-response curves using GraphPad Prism. The lowest concentrations at which cardiac contractile function or cell viability registered significantly below that of the solvent control were also identified with one-way analysis of variance (ANOVA) statistical analysis and a subsequent post hoc test as appropriate. *p*-values below 0.05 were considered significant.

## 5. Conclusions

In summary, this proof-of-concept study has laid the groundwork for further developing a cardiac EST embryotoxicity evaluation protocol based on human iPSCs and fibroblasts modeled after the original cardiac mouse EST. Incorporating early tissue marker endpoints as outlined in this protocol also possibly offers an opportunity to reduce the time commitments surrounding traditional animal embryotoxicity screens and the in vitro contractile assay to increase throughput with an opening for automated assessments and reduced culture time.

## Figures and Tables

**Table 1:** Comparison of mESC- and hiPSC-EST IC_50_ and ID_50_ values and embryotoxicity classifications.

Cell Type	Toxicity Parameter	Penicillin G	5-Fluorouracil	*All-Trans* Retinoic Acid
mESCs	ID_50_ (μg/mL)	750	9.7 × 10^−3^	1.7 × 10^−3^
	IC_50_ (μg/mL)	800	2.2 × 10^−2^	9 × 10^−5^
	3T3 IC_50_ (μg/mL)	1000	5 × 10^−2^	2.7
hiPSC	ID_50_ (μg/mL)	739.9	2.29 × 10^−6^	9.33 × 10^−7^
	IC_50_ (μg/mL)	<1000	4.7 × 10^−5^	3.642
	3T3 IC_50_ (μg/mL)	1433	8.1 × 10^−4^	1.25 × 10^−2^
Cmax (μg/mL)		134.6 [12]	0.94 [13]	0.155–0.294 [13]
mESC Biostatistical Embryotoxicity Classification		Not embryotoxic	Strongly embryotoxic	Strongly embryotoxic
hiPSC (day 25) Biostatistical Embryotoxicity Classification		Not embryotoxic	Strongly embryotoxic	Strongly embryotoxic

Half-maximal inhibitory doses determined with mESCs were previously obtained in our lab [14]. Inhibition of differentiation (ID_50_) in hiPSCs was determined from dose–response curves as 50% inhibition of functional cardiomyocytes in the control. Inhibition of cell viability (IC_50_) in hiPSCs was determined from dose–response curves as 50% inhibition of viable cells in control cultures. hiPSC-EST correctly classified all-trans retinoic acid and 5-flurouracil as embryotoxic. Cmax, maximum plasma concentration; mESC, murine embryonic stem cell; hiPSC, human induced pluripotent stem cell; EST, embryonic stem cell test.

**Table 2:** List of IC_50_ and ID_50_ values and embryotoxicity classifications determined from concentration-response curves for contractile and d10 qPCR assay endpoints. hiPSC, human induced pluripotent stem cell; hFF, human foreskin fibroblast.

Cell Type	Toxicity Parameter	Penicillin G	5-Fluorouracil	*All-Trans* Retinoic Acid
hiPSCs (d25)	ID_50_ (μg/mL)	739.9	2.29 × 10^−6^	9.33 × 10^−7^
	IC_50_ (μg/mL)	>1000	4.726 × 10^−5^	3.642
hiPSCs (d10 *TBX5*)	ID_50_ (μg/mL)	>1000	1.79 × 10^−4^	3.28 × 10^−6^
hFF	IC_50_ (μg/mL)	1433	8.079 × 10^−4^	1.25 × 10^−2^
hiPSC (day 25 cluster) Biostatistical Embryotoxicity Classification		Not embryotoxic	Strongly embryotoxic	Strongly embryotoxic
hiPSC (day 10 qPCR) Biostatistical Embryotoxicity Classification		Not embryotoxic	Strongly embryotoxic	Strongly embryotoxic

**Table 3:** List of IC_50_ and ID_50_ values determined from concentration-response curves for mainstream cigarette smoke and Snus smokeless tobacco. hiPSC, human induced pluripotent stem cell; hFF, human foreskin fibroblast.

Toxicity Parameter	Marlboro Red 100 MS (PE)	Nicotine Concentration at Half-Maximal Concentration Marlboro Red 100 MS (µM)	Camel Snus (% *w*/*v*)	Nicotine Concentration at Half-Maximal Concentration Samel Snus (µM)
hiPSC IC_50_	>0.1	6.1	>1	314.2
hiPSC ID_50_	0.014 ± 0.0047	0.085	0.0048	15.27
hFF IC_50_	1.367	82.41	0.2844	89.36
Biostatistical Embryotoxicity Classification	Strongly embryotoxic		Strongly embryotoxic	

## Data Availability

The data underlying this article are available in the article.
